# Prognostic biomarkers for immunotherapy in esophageal cancer

**DOI:** 10.3389/fimmu.2024.1420399

**Published:** 2024-09-30

**Authors:** Xu Tong, Meiyuan Jin, Lulu Wang, Dongli Zhang, Yuping Yin, Qian Shen

**Affiliations:** ^1^ Department of Gastrointestinal Surgery, Union Hospital, Tongji Medical College, Huazhong University of Science and Technology, Wuhan, China; ^2^ Tongji Medical College, Huazhong University of Science and Technology, Wuhan, China; ^3^ Tianjin Key Laboratory of Technologies Enabling Development of Clinical Therapeutics and Diagnostics, School of Pharmacy, Tianjin Medical University, Tianjin, China; ^4^ Department of Oncology, Tongji Hospital, Tongji Medical College, Huazhong University of Science and Technology, Wuhan, China

**Keywords:** esophageal cancer, immunotherapy, biomarkers, ICIs, TIME

## Abstract

Esophageal cancer (EC), a common type of malignant tumor, ranks as the sixth highest contributor to cancer-related mortality worldwide. Due to the condition that most patients with EC are diagnosed at advanced or metastatic status, the efficacy of conventional treatments including surgery, chemotherapy and radiotherapy is limited, resulting in a dismal 5-year overall survival rate. In recent years, the application of immune checkpoint inhibitors (ICIs) has presented a novel therapeutic avenue for EC patients. Both ICIs monotherapy and immunotherapy combined with chemotherapy or chemoradiotherapy (CRT) have demonstrated marked benefits for patients with advanced EC. Adjuvant or neoadjuvant therapy incorporating immunotherapy has also demonstrated promising prospects in the context of perioperative treatment. Nonetheless, due to the variable response observed among patients undergoing immunotherapy, it is of vital importance to identify predictive biomarkers for patient stratification, to facilitate identification of subgroups who may derive greater benefits from immunotherapy. In this review, we summarize validated or potential biomarkers for immunotherapy in EC in three dimensions: tumor-cell-associated biomarkers, tumor-immune microenvironment (TIME)-associated factors, and host-associated biomarkers, so as to provide a theoretical foundation to inform tailored therapy for individuals diagnosed with EC.

## 1 Introduction

Esophageal cancer (EC), including esophageal squamous cell carcinoma (ESCC) and esophageal adenocarcinoma (EAC), ranks as the eighth most common malignancy worldwide, with a mortality rate ranking sixth (1, 2). In China, EC, predominantly consisting of ESCC cases, poses a substantial disease burden, accounting for approximately half of the global annual incidence and mortality rates (3). Anatomically, ESCC mainly occurs in the upper or middle segment of the esophagus, whereas EAC tends to manifest in the distal region (4). Geographically, ESCC is more commonly found in Eastern Asia, Eastern Europe, and Southern and Eastern Africa, while EAC has a higher prevalence in North America, Central America, and Central Africa (3, 4).

**Figure 1:**
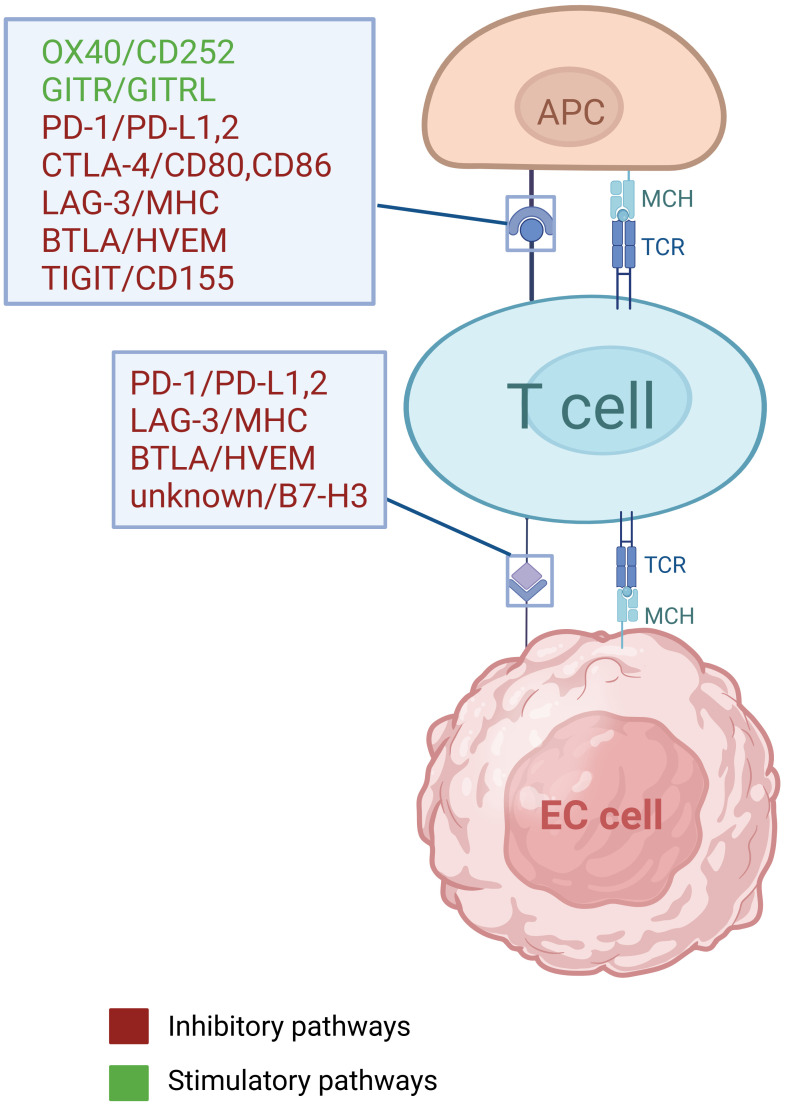
Immune checkpoint genes and cellular interactions contributing to tumor immunity.

Conventional treatment strategies for EC include surgery, radiotherapy, chemotherapy and targeted therapy (5), but the five-year survival rate still remained less than 20% until 2021, due to delayed diagnoses and high recurrence rates (3, 4). In recent years, the administration of immune checkpoint inhibitors (ICIs) in EC has attained significant advances, as ICIs can both suppress cancer cells by modulating anti-tumor immunity, as well as exerting a synergistic effects when combined with chemotherapy or/and radiotherapy (6) (**Table 1**). Immune checkpoint genes and cellular interactions contributing to tumor immunity are illustrated in **Figure 1** (7, 8) (**Figure 1**). The phase III KEYNOTE-181 trial demonstrated that pembrolizumab, compared with chemotherapy, contributed to superior overall survival (OS), objective response rate (ORR), and lower incidence of high-grade treatment-related adverse events (9). Moreover, other phase III trials, such as CheckMate648 (10) and KEYNOTE-590 (11), have confirmed that the combination of immunotherapy and chemotherapy as first-line treatment significantly improves OS and progression-free survival (PFS) compared with chemotherapy alone. However, the benefits of immunotherapy vary among patients with patients, highlighting the significance of identifying reliable biomarkers that can predict their response to immunotherapy.

**Table 1:** The large-scale phase III clinical trials of immunotherapy for EC and efficacy outcomes in overall population regardless of PD-L1 expression.

Clinical trial	Treatment model	Cancer status	Region	Arm (No. of pts)	Treatment design	Efficacy outcomes (95% CI)	Ref.
KEYNOTE-590 (NCT03189719)	First-line	M/UA EC	global	1 (373)	PEM (200 mg) + chemotherapy	Improved OS: 12.4 (10.5 - 14.0) m; Improved PFS: 6.3 (6.2 - 6.9) m; Improved ORR: 45.0 (39.9 - 50.2) %	(11)
				2 (376)	placebo + chemotherapy	OS: 9.8 (8.8 - 10.8) m; PFS: 5.8 (5.0 - 6.0) m; ORR: 29.3 (24.7 - 34.1) %	
CheckMate 648 (NCT03143153)	First-line	M/UA ESCC	global	1 (321)	NIV (240 mg) + chemotherapy	Improved OS: 13.2 (11.1 - 15.7) m; PFS: 5.8 (5.6 - 7.0) m	(10)
				2 (325)	NIV (3 mg/kg) + IPI (1 mg/kg)	Improved OS: 12.7 (11.3 -15.5) m; PFS: NA	
				3 (324)	Chemotherapy	OS: 10.7 (9.4 - 11.9) m; PFS: 5.6 (4.3 - 5.9) m	
ESCORT-1st (NCT03691090)	First-line	A/M ESCC	China	1 (298)	CAM (200 mg) + chemotherapy	Improved OS: 15.3 (12.8 - 17.3) m; Improved PFS: 6.9 (5.8 - 7.4) m; Improved ORR: 72.1 (66.7 -77.2) %	(12)
				2 (298)	placebo + chemotherapy	OS: 12.0 (11.0 - 13.3) m; PFS: 5.6 (5.5 - 5.7) m; ORR: 62.1 (56.3 - 67.6) %	
ORIENT-15 (NCT03748134)	First-line	LA/M ESCC	global	1 (327)	SIN (3 mg/kg or 200 mg) + chemotherapy	Improved OS: 16.7 (14.8 - 21.7) m; Improved PFS: 7.2 (7.0 - 9.5) m; Improved ORR: 66 (61– 71) %	(13)
				2 (332)	placebo + chemotherapy	OS: 12.5 (11.0 - 14.5) m; PFS: 5.7 (5.5 - 6.8) m; ORR: 45 (40 –51) %	
JUPITER-06 (NCT03829969)	First-line	LA/M ESCC	China	1 (257)	TOR (240 mg) + chemotherapy	Improved OS: 17.0 (14 - NA) m; Improved PFS: 5.7 (5.6 - 7.0) m; Improved ORR: 69.3 (63.2 - 74.8) %	(14)
				2 (257)	placebo + chemotherapy	OS: 11.0 (10.4 -12.6) m; PFS: 5.5 (5.2 - 5.6) m; ORR: 52.1 (45.8 - 58.4) %	
RATIONALE-306 (NCT03783442)	First-line	LA/M ESCC	global	1 (326)	TIS (200 mg) + chemotherapy	Improved OS: 17.2 (15.8 -20.1) m; Improved PFS: 7.3 (6.9 - 8.3) m; Improved ORR: 63 (58 – 69) %	(15)
				2 (323)	placebo + chemotherapy	OS: 10.6 (9.3 - 12.1) m; PFS: 5.6 (4.9 -6.0) m; ORR: 42 (37 – 48) %	
KEYNOTE-181 (NCT02564263)	Second-line	LA/M EC	global	1 (314)	PEM (200 mg)	OS: 7.1 (6.2 - 8.1) m; PFS: 2.1 (2.1 - 2.2) m; Improved ORR: 13.1 (9.5 - 17.3) %	(9)
				2 (314)	chemotherapy	OS: 7.1 (6.3 - 8.0) m; PFS: 3.4 (2.8 - 3.9) m; ORR: 6.7 (4.2 - 10.0) %	
ESCORT (NCT03099382)	Second-line	A/M ESCC	China	1 (228)	CAM (200 mg)	Improved OS: 8.3 (6.8 - 9.7) m; Improved PFS: 1.9 (1.9 - 2.4) m; Improved ORR: 20.2 (15.2 - 26.0) %	(16)
				2 (220)	chemotherapy	OS: 6.2 (5.7 - 6.9) m; PFS: 1.9 (1.9 - 2.1) m; ORR: 6.4 (3.5 - 10.5) %	
RATIONALE-302 (NCT03430843)	Second-line	LA/M ESCC	global	1 (256)	TIS (200 mg)	Improved OS: 8.6 (7.5 - 10.4) m; PFS: 1.6 (1.4 - 2.7) m; Improved ORR: 20.3 (15.6 - 25.8) %	(17)
				2 (256)	chemotherapy	OS: 6.3 (5.3 - 7.0) m; PFS: 2.1 (1.5 - 2.7) m; ORR: 9.8 (6.4 - 14.1) %	
ATTRATION-3 (NCT02569242)	Second-line	A/R ESCC	global	1 (210)	NIV (240 mg)	Improved OS: 10.9 (9.2 - 13.3) m; PFS: 1.7 (1.5 - 2.2) m; ORR: 19.3 (14 – 26) %	(18)
				2 (209)	chemotherapy	OS: 8.4 (7.2 - 9.9) m; PFS: 3.4 (3.0 - 4.2) m; ORR: 21.5 (15 – 29) %	

A, advanced; CAM, camrelizumab; IPI, ipilimumab; LA, locally advanced; M, metastatic; m, months; NA, not available; NIV, Nivolumab; ORR, objective response rate; OS, overall survival; PEM, pembrolizumab; PFS, progression-free survival; pts, patients; R, refractory; SIN, sintilimab; TIS, tislelizumab; TOR, toripalimab; UA, unresectable advanced.

Based on the latest advances in the field, this paper review categorizes prognostic biomarkers for patients with EC into three facets: tumor intrinsic factors, tumor-immune microenvironment (TIME)-associated factors, and host-related factors. Further research into these biomarkers, elucidating their mechanisms in mediating anti-tumor immunity and clarifying their clinical significance in EC immunotherapy, may aid in the identification of populations likely to benefit or in searching therapeutic targets that can enhance ICIs efficacy.

## 2 Tumor-cell-associated biomarkers

Tumor-cell-associated biomarkers, including tumor mutation burden (TMB) and high microsatellite instability (MSI-H), are established prognostic indicators for EC immunotherapy, while the evidence for programmed cell death ligand 1 (PD-L1) as a marker in this context has led to disputed conclusions (6). In this section, we summarize clinical findings relating both to these established biomarkers and to emerging markers, and explore the mechanisms potentially underlying related clinical phenomena.

### 2.1 PD-L1

The level of PD-L1 expression, generally assessed by combined positive score (CPS) or tumor proportion score (TPS), stands as a prognostic biomarker for predicting the efficacy of PD-1/PD-L1 inhibitors (19, 20). Many studies have reported correlations between elevated PD-L1 expression and adverse prognosis in patients with EC, including lymph node and distant metastasis, as well as poor OS (20). For example, Yagi et al. observed that high PD-L1 expression in patients with surgically resected EC indicated higher recurrence rate and shorter OS (21). The American Society of Clinical Oncology has issued a guideline pertaining to the use of immunotherapy for advanced gastroesophageal cancer, emphasizing the importance of PD-L1 testing and mismatch repair status assessment (22), which suggests conducting biomarker testing for individuals diagnosed with EC and gastroesophageal junction cancer, and formulating therapy models based on the results of CPS and TPS (22).

Although high PD-L1 expression tends to suggest the poor prognosis, some studies have shown that PD-1/PD-L1 inhibitors yield better therapeutic effects in patients with raised PD-L1 expression. In KEYNOTE-590 trial, pembrolizumab plus chemotherapy improved OS (p < 0.001) and PFS (p < 0.001) in ESCC participants with PD-L1 CPS ≥ 10 versus chemotherapy alone (11). CheckMate 648 study obtained similar results, showing that patients with elevated PD-L1 expression had superior OS and PFS after nivolumab treatment compared with the overall population (10). Nevertheless, clinical trials, such as ESCORT-1st (12), ORIENT-15 (13), and Jupiter-06 (14), have reported results indicating that PD-1 blockade agent efficacy is not correlated with PD-L1 level. In other words, immunotherapy plus chemotherapy was beneficial for patient OS regardless of PD-L1 status (**Table 2**). Therefore, whether PD-L1 can be considered a prognostic biomarker for immunotherapy in EC remains controversial.

**Table 2:** Prognostic value of elevated PD-L1 expression in immunotherapy for EC.

Clinical trial	Phase	Pathological type	Status/stage	ICIs Target	Treatment model	Detection method	Cut-off point	Prognostic value	Ref.
KEYNOTE-590 (NCT03189719)	3	EC	M/UA	PD-1	First-line	IHC 22C3	CPS: 10	Improved OS; Improved PFS	(11)
CheckMate 648 (NCT03143153)	3	ESCC	M/UA	PD-1	First-line	IHC 28-8	TPS: 1%	Improved OS; Improved PFS	(10)
ESCORT-1st (NCT03691090)	3	ESCC	A/M	PD-1	First-line	6E8 antibody	TPS: 1%	NSS	(12)
ORIENT-15 (NCT03748134)	3	ESCC	LA/M	PD-1	First-line	IHC 22C3	CPS: 1; 5; 10 TPS: 1%; 5%; 10%	NSS (prolonged duration of confirmed response, p = NA)	(13)
JUPITER-06 (NCT03829969)	3	ESCC	LA/M	PD-1	First-line	IHC JS311	CPS: 1; 10	NSS	(14)
RATIONALE-306 (NCT03783442)	3	ESCC	LA/M	PD-1	First-line	VENTANA SP263	TAP: 10%	NSS	(15)
EC-CRT-001 (NCT04005170)	2	ESCC	LA	PD-1	First-line	IHC 22C3	CPS: 10	NSS (improved CRR but p = 0.52)	(23)
KEYNOTE-181 (NCT02564263)	3	EC	LA/M	PD-1	Second-line	IHC 22C3	CPS: 10	Superior to chemotherapy	(9)
ESCORT (NCT03099382)	3	ESCC	A/M	PD-1	Second-line	6E8 antibody	TPS: 1%; 5%; 10%	NSS (improved OS but p = 0.13)	(16)
RATIONALE-302 (NCT03430843)	3	ESCC	LA/M	PD-1	Second-line	VENTANA SP263	TAP: 10%	NSS (improved OS but p = 0.21)	(17)
NCT02971956	2	EAC	A	PD-1	At least second-line	IHC 22C3	CPS: 1; 10	NSS (improved OS but p = 0.28; Improved PFS but p = 0.22)	(28)
NCT02730546	1b/2	AEG	cT_1–3_ N_any_ M_0_	PD-1	NAT	IHC 22C3	CPS: 1; 10	Improved pCR	(29)
ChiCTR- 1900026240	2	ESCC	Stage III/IVa	PD-1	NAT	IHC 22C3	CPS: 1 TPS: 50%	NSS	(30)
NCT04177797	2	ESCC	LA; Stage III/IVa	PD-1	NAT	IHC 22C3	CPS: 1 TPS: 1%	NSS	(31)
PERFECT (NCT03087864)	2	EAC	<cT_4b_N_0_ or cN_+_M_0_	PD-L1	NAT	IHC 22C3	CPS: 1; 10; 25	NSS	(32)
NCT02520453	2	ESCC	Stage II/III	PD-L1	NAT	VENTANA SP263	TPS: 1%	NSS (improved OS but p = 0.18; Improved DFS but p = 0.54)	(24)

A, advanced; AEG, adenocarcinoma of esophagogastric junction; CRR, complete response rate; DFS, disease-free survival; LA, locally advanced; M, metastatic; NA, not available; NAT, neoadjuvant therapy; NSS, not statistically significant; pCR, pathological complete response; TAP, tumor area positivity, UA, unresectable advanced.

Notably, some studies have reported that patients with heightened PD-L1 expression may exhibit favorable complete response rate (CRR), disease-free survival (DFS) and OS, but the statistical differences were not significant (p > 0.05) (23, 24). These contradicting findings regarding PD-L1 may be attributable to various factors including: 1) disease heterogeneity, such as variation in pathological types and stages; 2) differences in the ICIs administered; 3) variations in PD-L1 detection methods; 4) discrepancies in cut-off points; 5) inconsistency in the timing of PD-L1 detection (i.e., baseline versus post-treatment); 6) variations in sample sizes (25, 26). In the future, normalization and standardization of PD-L1 detection may contribute to clarifying its relationship with ICIs efficacy (27).

### 2.2 TMB

TMB, characterized by the frequency of somatic mutations in the coding regions of tumor genomes, has emerged as a prognostic biomarker for ICIs therapy in various types of cancer, including EC (33–35). After transcription and translation, mutations in tumors generate neoantigens, which increase the immunogenicity of tumor cells and thus elicits an intenser anti-tumor immune activation induced by ICIs (34). Huang et al. found that ESCC patients with higher TMB (more than 60 missense mutations) showed improved clinical benefits in response to camrelizumab remedy (36). The KEYNOTE-158 study confirmed that patients with elevated TMB status (more than 10 mutations per megabase) in advanced solid tumors who received pembrolizumab had a significantly higher ORR than other patients (33), which led FDA to grant accelerated endorsement for the use of pembrolizumab for managing unresectable or metastatic solid tumors with TMB above 10 Mut/Mb in 2020 (37).

Tumors with enhanced TMB tend to generate a greater density of neoantigens, thereby inducing anti-tumor immune response characterized by recognition and cytotoxicity (38), which can be enhanced when ICIs block immune checkpoints to facilitate anti-tumor immunity. A multiomics analysis revealed a correlation between TMB and mismatch repair (MMR) status, as well as the infiltration of immune cells such as regulatory T cells (Tregs), monocytes, and T helper cells (Ths) in EC (39). Another bioinformatics analysis identified a positive correlation between nonsynonymous TMB level and the infiltration of resting NK cells (p = 0.028), Tregs (p = 0.064), and CD8^+^ T cells (p = 0.12) (40). Furthermore, elevated TMB also suggests a shorter distance between EC cells, especially PD-L1^-^ tumor cells, to DCs and macrophages, which is associated with improved OS and PFS (41). This suggests that an increase in TMB leads to the distribution of antigen-presenting cells around the proximal tumor cells, thereby ameliorating the prognosis of patients undergoing immunotherapy.

### 2.3 MSI

MSI refers to the variations in the length of tandem repeats caused by defects in the DNA MMR system (42). Generally, tumors in which more than 30–40% of markers are mutated are termed as MSI-high (43). In a clinical study involving various types of solid malignancy, including gastroesophageal cancers, patients afflicted with MMR-deficient cancers and receiving pembrolizumab treatment exhibited favorable outcomes, with 53% achieving objective radiographic responses and 21% experiencing complete responses (44), indicating that ICIs are highly effective in the treatment of MSI-high patients regardless of the origin of the tumor tissue (44). Hence the FDA authorized the application of pembrolizumab in combating MSI-H solid tumors, marking the first time that the FDA has established a biomarker for treatment without restriction based on tumor type (45, 46).

Similar to the mechanism of TMB, MSI-H tumor cells generate more neoantigens, which in turn augments T cells’ proliferation and activation, making such tumors more susceptible to anti-tumor immune responses (44, 45). A study of adenocarcinoma of esophagogastric junction (AEG) demonstrated that MSI-H tumors were associated with elevated level of CD8^+^ T cells infiltration in both the intratumoral region and invasive margin, as well as higher PD-L1 expression level in AEG cells, compared with microsatellite stable (MSS) tumors (47). By contrast, although MSI-low tumors also showed increased level of CD8^+^ T cells in intratumoral area, no differences were observed in the invasive margin or in PD-L1 expression compared to MSS tumors (47). Similar results have also been observed in MSI-H colorectal cancer, which produces more neopeptides that are more readily recognized by the immune system than MSI-low tumor, and therefore eliciting an increased infiltration level of Th1 cells and CD8^+^ T cells (48).

### 2.4 Abnormal DNA methylation

DNA methylation is a common epigenetic modification, characterized in mammals by the methylation of the C5 position of cytosine to form 5-methylcytosine (49). Aberrant DNA methylation is a significant contributor to tumorigenesis and progression, and can serve as a prognostic biomarker for ESCC (50). For example, higher methylation levels in the promoters of miR129-2 and miR124-3 are associated with inferior response to neoadjuvant CRT of EC patients (51). However, studies discussing the relationship between DNA methylation and prognosis in the context of ICIs treatment are relatively limited. One study stratified 94 ESCC patients into two subgroups, S1 (n = 40) and S2 (n = 54), based on their DNA methylation and gene expression data (52). Genes differentially expressed between the two subgroups were identified as primarily involved in immune system regulation, immune cell activation, and cytokine function, according to KEGG/GO analysis. Taking their DNA methylation or gene expression data as the training set, the authors established a linear SVM model comprising 15 genes. Analysis of validation set data from 36 ESCC patients treated with PD-1/PD-L1 blockades plus chemotherapy was assembled. The final results demonstrated that the 15-gene expression signature exhibited a sensitivity of 68.8%, a specificity of 75%, and an efficacy of 72.2% in predicting responses to immunotherapy (52).

As mentioned previously, differences in DNA methylation and gene expression influenced the activation and immunological function of a wide range of immune cells in the S1 and S2 groups. The researchers investigated the condition of tumor microenvironment (TME) in each of the samples, observing that the patients in S2 group had higher levels of Tregs, resting memory CD4^+^ T cells, Ths, macrophages and activated mast cells, as well as higher levels of immune checkpoint molecules, including CTLA-4 and lymphocyte activation gene-3 (LAG-3) (52). These findings indicate that aberrant DNA methylation can result in T cell exhaustion and suppression of anti-tumor immunity. Given the significant role of DNA methylation in immune responses against cancers, it is anticipated to be a valuable biomarker for EC immunotherapy (53).

### 2.5 Amplification of chromosome 11q13

The amplification of genomic region in chromosome 11q13 is among the most common aberrations observed in various malignant tumors, including ESCC (54). Chromosome 11q13 amplification contributes to promoting lymphangiogenesis, lymphatic metastasis, and suppression of immune response within the TME, thus hindering anti-cancer therapies (54, 55). In a clinical study involving advanced ESCC patients undergoing toripalimab therapy, those without 11q13 amplification (n=26) demonstrated significantly higher ORR (p = 0.024) and longer PFS (p = 0.025) than those with 11q13 amplification (n=24) (56), suggesting that chromosome 11q13 amplification may serve as an unfavorable biomarker for ESCC treated with PD-1 inhibitors (56), and similar result were observed in unresectable hepatocellular carcinoma (HCC) (57).

Chromosome 11q13 amplification includes the amplification of miR-548k and Cyclin D1 (CCND1), among others (54, 55, 58). MiR-548k promotes lymphangiogenesis by stimulating VEGFC secretion and activating ADAMTS1/VEGFC/VEGFR3 signaling pathway (54). Additionally, MiR-548k facilitates nodal metastasis by regulating the LF10/EGFR axis (54). CCND1 amplification leads to a state of immune exhaustion in the TME, which manifests as decreased densities of CD8^+^ T cells, dendritic cells (DCs), and B cells and an elevated levels of Ths, Tregs, and myeloid-derived suppressor cells (MDSCs) (55). CCND1 exerts its function by activating CDK4/6 (57), and it is reported that the combined use of CDK4/6 inhibitors and anti-PD-1 antibodies significantly improves the survival outcomes in a mouse model of colon cancer (59). Moreover, chromosome 11q13 amplification is accompanied by an increased density of Foxp3^+^ Tregs, which may contribute to hyperprogressive disease, a condition characterized by primary resistance to immunotherapy (57).

### 2.6 Amplification of MCL-1

Myeloid cell leukemia 1 (MCL-1) was isolated from a human myeloid leukemia cell line and belongs to the Bcl-2 protein family (60). MCL-1 amplification and overexpression is associated with the proliferation, drug resistance and inferior prognosis in various tumors including ESCC (61, 62), and inhibition of CPEB4-mediated MCL-1 translation can reverse the resistance to cisplatin in ESCC (63). Both MCL-1 and Bcl-xL belong to the bcl-2 family (60). Combination therapy targeting MCL-1 and Bcl-xL effectively eliminates melanoma cells from patients who experience relapse after PD-1 or CTLA-4 remedy, and curtails the self-renewal capability of melanoma cells (64).

In EC-CRT-001 trial involving ESCC patients treated with toripalimab plus CRT, univariable analyses of 16 genes of interest were conducted and only MCL-1 level was significantly associated with shorter OS (p = 0.03) and PFS (p = 0.024) (23). In *post-hoc* analysis, it emerged that patients with MCL-1 amplification demonstrated elevated levels of PD-L1^+^ CD8^+^ T cells and PD-L1^+^ macrophages infiltration (23). In addition, a pan-cancer study found that the NANOG/HDAC1/MCL-1 axis mediates tumor resistance to PD-1 inhibitors, which could be reversed by silencing MCL-1 (65, 66). Collectively, these studies indicate a significant role for MCL-1 in mediating the immune-refractory state, thus suggesting the potential value of MCL-1 as both a biomarker and a therapeutic target in EC immunotherapy.

### 2.7 Long non-coding RNA (lncRNA)

long non-coding RNAs (lncRNAs), which is associated with tumor progression and immune evasion (67, 68), can be transcribed from both coding regions and regulatory regions of the tumor itself (69), or be delivered into cancer cells by TAMs via exosomes (70). Enhancers, as critical components of regulatory regions, are activated through demethylation and are subsequently transcribed into enhancer RNAs (eRNAs), a type of lncRNAs that are involved in the upregulation of corresponding target genes (71). Gao et al. constructed an EDRGS score model based on 12 target genes in ESCC and found that patients with higher EDRGS exhibited superior responses to anti-PD-1 regimen (p = 0.038) (71). Bulk RNA-seq and scRNA-seq analyses revealed that EDRGS-high group exhibited elevated infiltration of CD8^+^ T cells and NK cells, as well as upregulated levels of PD-1, LAG-3, TIM3, and TIGIT. In other words, EDRGS-high group denoted an immune-hot but immune-suppressive phenotype, accounting for improved response to ICIs (71). Additionally, it was found that eRNA AC005515.1 is co-expressed with several immune checkpoint genes, including CTLA4, Foxp3, and IDO1, and is positively correlated with the infiltration of CD8^+^ T cells and M1 macrophages (72). Interestingly, patients with higher expression of AC005515.1 had increased TIDE scores and worse survival outcomes (72). This bioinformatics-based inference appears to be inconsistent with Gao’s finding, highlighting the importance of further experiments and clinical studies to verify these results.

Another lncRNA, LINC02096, has been identified as a biomarker for ESCC immunotherapy by regulating immune evasion (73). Patients with elevated level of LINC02096 demonstrated inferior disease control rate (DCR) and ORR when undergoing anti-PD-1 monotherapy. In mouse model, knockdown of LINC02096 in TAMs upregulated the level of cytotoxic CD8^+^ T cells, which rescued tumor progression and enhanced the treatment efficacy of anti-PD-1 antibody (73). Further research confirmed that high levels of LINC02096 are accumulated in ESCC cells with the involvement of exosomes secreted by TAMs and TNF-α. In tumor cells, LINC02096 inhibits the ubiquitination of histone methyltransferase MLL1, which enhances the levels of H3K4me3 in the promoter regions of PD-L1 and IDO-1, thereby undermining anti-tumor immunity (73).

## 3 Tumor-immune-microenvironment-associated biomarkers

The whole tumor-immune microenvironment (TIME) can be viewed as a biomarker for immunotherapy. TIME-associated biomarkers encompass various components, such as tumor-infiltrating lymphocytes (TILs), tumor-associated macrophages (TAMs), and cytokines (74).

### 3.1 TILs

#### 3.1.1 Conventional immune biomarkers

A meta-analysis has confirmed that TILs overall can serve as a prognostic biomarker for OS in patients with EC (75). For surgically resected EC, patients with TIL-positive status tend to achieve favorable OS and DFS (21). However, treatments in the analyzed studies were not solely confined to ICIs therapy. Additionally, there is evidence that different TILs subsets may have distinct prognostic values (75). Therefore, the predictive role of TILs in EC immunotherapy warrants further elucidation.

In a clinical trial involving ESCC patients undergoing tislelizumab plus chemotherapy followed by esophagectomy, levels of CD8^+^ T cells, Ths, Tregs, and mature DCs were significantly increased in pathological complete response (pCR) group, while the density of B cells and neutrophils significantly decreased (76). Another clinical study of locally advanced ESCC also detected a statistically significant correlation between CD8^+^ T cells content, rather than other TIL subsets, and clinical response, with improved CRR (p = 0.004) and prolonged PFS (p = 0.005) (23). Another trial researching refractory EC demonstrated that patients with more abundant PD-1^+^ CD4^+^ T cells exhibited poorer radiological response (p = 0.035), and that the expression of PD-1 and T-cell immunoglobulin and mucin domain-3 (TIM-3) on CD4^+^ T cells suggested early progression (28). We consider that the infiltration of a certain quantity of CD8^+^ T cells is necessary for a physiological anti-tumor immune response; however, given the complex functions of diverse immune cells, how some immune cell types exert negative effects on EC immunotherapy requires further investigation.

#### 3.1.2 Novel immune biomarkers

Novel immunotherapeutic targets have emerged in recent years, including LAG-3 and TIM-3, among others (5). However, due to the limited availability of clinical results related to these molecules, it is currently premature to reach conclusions regarding these factors. Therefore, we have included them as potential biomarkers and summarized relevant findings in the Discussion section of this review. TCF-1 expressed on CD8^+^ T cells may serve as a novel immune biomarker. A study investigating pembrolizumab combined with CRT in ESCC observed that patients in the pCR group exhibited a higher level of TCF-1^+^ cells than those in the non-pCR group (p = 0.01) (77). In melanoma, TCF-1^+^ CD8^+^ T cells are considered a positive biomarker, which will proliferate, self-renew, or differentiate into TCF-1^-^ CD8^+^ T cells after treatment with ICIs (78). TCF-1 plays essential roles in maintaining the stem-like function of CD8^+^ T cells and in intratumoral immune responses (79). Even when the influx of new T cells is blocked, melanoma mice with elevated TCF-1^+^ TILs can still control tumor growth. Conversely, when TCF-1^+^ TILs are suppressed, tumor control ability is lost (79).

### 3.2 TAMs

TAMs are immune cells with crucial roles in the TIME, whose density, distribution, and subtypes are correlated with the prognosis of EC (80–82). In a clinical trial investigating toripalimab plus chemotherapy in patients with locally advanced ESCC, responders showed a lower density of M2-TAMs (31). Another study involving camrelizumab plus CRT demonstrated that higher density of PD-L1^-^ macrophages in the baseline tumor compartment was associated with improved PFS (p = 0.032) and in the on-treatment compartment indicated superior OS (p = 0.018) and PFS (p = 0.028) (41, 83). Through spatial multi-immunofluorescence, it was found that PD-L1^-^ macrophages are situated closer to tumor cells than PD-L1^+^ macrophages, which may account for better clinical outcomes, to some extent (41). This study also found that the spatial distribution of TAMs was correlated with TMB, while previous reports have confirmed positive correlation between TAMs and PD-L1 levels in EC (84), and shown that TAMs could also secrete a variety of chemokines to regulate the TIME (85, 86), suggesting that the prognostic significance of TAMs may need to be judged in conjunction with other biomarkers (84). Notably, this study did not differentiate between M1 and M2 TAMs, nor did it investigate any association between the expression of CD68 or CD163 on macrophages and immunotherapy efficacy, which are areas warranting further research.

### 3.3 Cytokines

#### 3.3.1 Interferon-γ (IFN-γ)

The PERFECT study tested for a six-gene IFN-γ signature in EAC patients receiving neoadjuvant immunotherapy based on previous experience and found that responders had higher levels of the baseline IFN-γ signature (p = 0.043) (32). Researchers conducted gene expression profile analysis of melanoma patients treated with pembrolizumab, and identified an IFN-γ-related signature as a prognostic marker by comparing responders and non-responders, further confirming its association with PFS and ORR in head and neck squamous cell carcinoma (HNSCC) and gastric cancer (GC) (87). These findings may be attributable to the involvement of IFN-γ in antigen presentation, chemokines secretion, and cytotoxicity, all of which are essential for the efficacy of immunotherapy (87). The upregulation of PD-L1 in the TME is established as partly originating from the effects of IFN-γ released by CD8^+^ T cells, indicating that IFN-γ can affect other biomarkers to indirectly influence immunotherapy (88).

#### 3.3.2 Chemokines

A study involving toripalimab plus chemotherapy in ESCC found that responders exhibited decreased CCL19 and elevated CXCL5 levels at baseline (31). CCL19 can activate the MEK1-ERK1/2 and PI3K-AKT pathways in M1-TAMs via CCR7, mediating their chemotaxis (89). However, although this finding regarding CXCL5 is consistent with the conclusion from a study of melanoma treated with nivolumab (90), the impact of CXCL5 on the TIME seems contradictory to its role as a positive biomarker, considering a study from skin cancers demonstrated that CXCL5 secreted by TAMs recruits MDSCs to the TME via the CXCR2-CXCL5 axis, exerting immunosuppressive effects (85). In addition, the infiltration of TAMs is reported to be modulated by the CCL2-CCR2 axis to affect PD-1 signaling pathway, leading to immune evasion (91), and CCL18 released by TAMs can promote tumor proliferation through activating the JAK2/STAT3 pathway (86), all of which contribute to the unsatisfactory prognosis of patients with ESCC.

## 4 Host-associated biomarkers

Host-associated biomarkers for cancer treatment include nutritional status (anemia, cachexia, etc.), peripheral blood immune substances, microbiomes, psychological disorders (including anxiety, depression, etc.), and endogenous hormones, among other factors. Promising results have been reported related to nutritional status, peripheral blood immune substances, antibiotic (ATB) use and its effects on microbiomes, and endogenous glucocorticoid in patients with EC.

### 4.1 Nutritional status

It is common for patients with upper gastrointestinal cancer to experience poor nutritional status, mainly due to reduced food intake and enhanced nutritional consumption by tumors (92). A recent retrospective study confirmed that the prognostic nutritional index (PNI) can serve as a biomarker for predicting the OS (p = 0.047) and PFS (p = 0.020) of EC patients undergoing anti-PD-1 inhibitors (93).

PNI, calculated from serum albumin content and total lymphocyte count, reflects the nutritional status and immunity of tumor patients (94). Albumin level reflects nutritional status, and a decrease in this factor indicates that the patient is in a state of malnutrition, which is a risk factor influencing prognosis (95). Lymphocytes exert anti-tumor immunity through cytokine-mediated cytotoxicity and insufficient lymphocytes results in lack of immune surveillance against tumors (96). Additionally, there are positive correlations between PNI and TILs status, CD8^+^ cells density and Foxp3^+^ cells density in EC patients, suggesting that lower PNI is associated with an unfavorable TIME, leading to poor response to immunotherapy (97).

### 4.2 Peripheral blood immune substances

Tumor-associated immunity can be categorized into immune responses within the TME and systemic immunity throughout the body, where the latter can be assessed by indicators such as neutrophil to lymphocyte ratio (NLR) and absolute neutrophil counts (ANC),among others (98). A real-world study of ESCC observed that an increase in NLR after ICIs treatment predicted decreased OS (p = 0.004) and inferior PFS (p = 0.019) (98). Similarly, the ORIENT-2 study showed that ESCC patients treated with sintilimab exhibited prolonged OS (p < 0.001) and PFS (p = 0.006) if the NLR was less than three at the sixth week (99). These findings may be attributable to the fact that neutrophils curtail the anti-tumor immunity mediated by NK cells and T cells, and secrete various cytokines, including IL-1 and IL-6, contributing to tumor proliferation (100–102).

Moreover, in neoadjuvant therapy combining toripalimab with chemotherapy for ESCC patients, although no relationship was observed between NLR and patients’ response, higher ANC and absolute natural killer cell counts were detected in responders (31). This phenomenon appears contradictory to the findings from patients with lung cancer, possibly due to the involvement of multiple chemotherapeutic agents in this study, which may have interfered with immunotherapy biomarker selection (103). Additionally, in ESCC patients treated with nivolumab, a rise in TIM-3^+^ CD4^+^ and TIM-3^+^ CD8^+^ T cells among peripheral blood mononuclear cells was observed in responders (104). Another study found that circulating CXCL10, interleukin 2 receptor α, and IL-6 were associated with pembrolizumab efficacy in treating EC, with favorable, unfavorable, and unfavorable relationships, respectively (28). These findings suggest that the prognostic significance of various immune substances in peripheral blood should not be neglected while TIME receives sufficient attention.

### 4.3 Antibiotic therapy and microbiomes

Microbiomes, including gut microbiota among others, can exert both protective and detrimental influences on cancer progression and therapeutic response (105). A meta-analysis incorporating retrospective studies of EC revealed that antibiotic (ATB) use from 60 days before to 30 days after ICIs treatment induced worse OS (P = 0.03) (106). This finding is consistent with the conclusion of a previous pan-cancer research (107), suggesting that ATB usage may lead to dysbiosis in patients’ microbiomes, affecting their immune function and response to ICIs (98, 106, 108, 109).

The relationships between gut microbiota and host immune function, tumor occurrence and progression, anti-tumor immunity, and patients’ prognosis are highly complex (105). Nevertheless, in general, the gut microbiota enhances antigen presentation mediated by DC cells, recruits and activates effector T cells to the TME, and reduces the density of Tregs and MDSCs (98), which provides a theoretical rationale for the beneficial role of normal gut microbiota in anti-tumor immunity. It is noteworthy that ATB use may not only affect patients’ prognosis by altering the composition and diversity of intestinal flora. Patients receiving ATB may have inherently compromised immune function and a state of infection or susceptibility to infection, which could also contribute to poor outcomes in immunotherapy. On the other hand, dysbiosis of the gut microbiota may not only be induced by ATB usage. A study investigating camrelizumab plus chemotherapy in ESCC revealed that patients experienced a decrease in gut microbiota diversity following treatment (110). Therefore, the relationship among ATB administration, alterations in both gut microbiota and intratumoral microbiomes, and response to immunotherapy warrants further investigation.

### 4.4 Endogenous glucocorticoid

Excessive glucocorticoid can suppress the proliferation and differentiation of naive T cells, inhibit the CD28-CD80/CD86 co-stimulatory pathway, and disrupt immune surveillance function by affecting tumor-infiltrating immune cells (111, 112). A retrospective study encompassing advanced or metastatic pan-cancer, including EC, found that patients with high baseline endogenous glucocorticoid levels tended to exhibit lower ORR (p < 0.001) and had poorer durable clinical benefits (DCB) (p = 0.001), as well as shorter OS (p < 0.001) and PFS (p < 0.001) (113). Further research indicated that elevated glucocorticoid level is associated with lower infiltration levels of lymphocytes, CD4^+^ T cells, and CD8^+^ T cells, as well as increased NLR (113).

In other malignancies, glucocorticoid-related signaling pathways are generally detrimental factors for immunotherapy (114, 115). In pancreatic ductal adenocarcinoma, researchers have confirmed that the glucocorticoid receptor (GR) can upregulate the expression of PD-L1 and downregulate the expression of MHC-1 in tumor cells, thereby impairing cytotoxic T cells’ infiltration and anti-tumor immune function (114). When GR is knocked down or inhibited, resistance to ICIs in mice will be reversed (114). In melanoma, HSD11B1 is an enzyme that can convert inert glucocorticoid into active glucocorticoid, and mice with high expression of HSD11B1 exhibit attenuated infiltration of CD4^+^ T cells and CD8^+^ T cells and are insensitive to PD-1 inhibitors (115). Blocking HSD11B1 leads to the decreased expression of CD206 and arginase-1 and the heightened expression of IL-12 in TAMs, as well as promoting secretion of IFN-γ by CD8^+^ T cells, thereby enhancing the efficacy of PD-1 blockades (115).

### 4.5 Circulating tumor DNA (ctDNA)

Circulating tumor DNA (ctDNA) refers to cell-free DNA present in body fluids such as blood, synovial fluid, and cerebrospinal fluid (116). As a non-invasive biomarker, ctDNA has been widely applied to treatment monitoring in recent years (116). While the ctDNA status before sintilimab treatment (i.e., the baseline ctDNA level) is not statistically significant with major pathological response (MPR) (p = 0.39), patients with undetectable ctDNA are more likely to achieve pCR (p = 0.008) (117). It is noteworthy that more attention is paid to the dynamic changes of ctDNA content compared to its baseline level (118). It was observed that patients with undetectable ctDNA or ctDNA clearance post-ICIs therapy tend to obtain prolonged recurrence-free survival (RFS) and OS (119). Similarly, the EC-CRT-001 trial found that patients with detectable ctDNA during or after treatment, rather than at baseline, exhibited shorter OS and PFS, as well as inferior clinical complete response (cCR) (120). ctDNA clearance represents neoantigen-specific T cell responses. In a clinical trial investigating neoadjuvant nivolumab alone or plus relatlimab treatment, two patients who achieved complete pathological response had ctDNA clearance following ICIs induction. Both of them exhibited expansions of neoantigen-specific T cell clones, which were not observed in patients with persistently detectable ctDNA (119). Currently, further studies are underway to explore the prognostic value of ctDNA in EC immunotherapy (121, 122).

## 5 Discussion

Despite variations in the most common pathological types across different geographical regions, EC poses significant threats and burdens to people worldwide (3). The emergence of ICIs has provided novel options for EC treatment, bringing promise for prolonged survival and improved quality of life for patients. ICIs are currently applied in first-line, second-line, adjuvant and neoadjuvant treatment patterns in EC (4, 123). However, not all EC patients can benefit equally from immunotherapy (5), highlighting the significance of identifying reliable biomarkers to discern subgroups who may respond better to immunotherapy. In this review we summarize tumor-cell-associated biomarkers, TIME-associated biomarkers, and host-associated biomarkers discovered in EC immunotherapy, as well as their possible underlying mechanisms (**Table 3**), with the goal of providing a theoretical basis for selection of suitable patients and administration of tailored treatments in the future.

**Table 3:** Summary of prognostic biomarkers for Immunotherapy in EC.

Biomarker	Source of evidence	ICIs target	Prognostic value*	Mechanism
Tumor-cell-associated biomarkers				
PD-L1	Clinical trial	PD-1	Improved OS; Improved PFS; Improved pCR; Superior to chemotherapy	PD-1 - PD-L1 axis is the therapeutic target of PD-1 inhibitors. Higher PD-L1 expression stands for the greater potential for ICIs efficacy
TMB & MSI	Clinical trial; Clinical samples; Bioinformatics analysis	PD-1	Improved ORR; Improved CRR; Improved CBR	Cancer cells with increased mutations produce more neoantigens, which stimulates anti-tumor immune response and mediates the infiltration of CD8^+^ T cells and the distance between APCs
Abnormal DNA methylation	Clinical samples; Bioinformatics analysis	PD-1; PD-L1	Unfavorable OS	S2 subgroup has an upregulated infiltration of Tregs, Ths, TAMs, activated mast cells and resting memory CD4^+^ T cells, representing a state of immune exhaustion and suppression
Chromosome 11q13 amplification	Clinical trial; Clinical samples; Cell lines; Mouse models; Bioinformatics analysis	PD-1; PD-L1; CTLA-4	Unfavorable PFS; Unfavorable ORR	Chromosome 11q13 amplification consists of the amplification of miR-548k and CCND1: 1) MiR-548k promotes lymphangiogenesis by ADAMTS1/VEGFC/VEGFR3 axis and facilitates nodal metastasis by LF10/EGFR pathway; 2) CCND1 induces the exhaustion of anti-tumor immune cells and elevates the density of Ths, Tregs, MDSCs
MCL-1 amplification	Clinical trial; Mouse models; Bioinformatics analysis	PD-1	Unfavorable OS; Unfavorable PFS	1) MCL-1 amplification is associated with augmented level of PD-L1^+^ CD8^+^ T cells’ and PD-L1^+^ macrophages’ infiltration; 2) NANOG/HDAC1/MCL-1 axis plays a pivotal role in displaying the resistant state against CTLs in TME
LncRNAs	Clinical samples; Cell lines; Mouse models; Bioinformatics analysis	PD-1	Unfavorable DCR; Unfavorable ORR	1) Influence the expression levels of target genes; 2) Elevate the expression levels of immune checkpoint genes, including PD-L1, IDO-1, LAG-3, TIM-3, etc. 3) Positively or negatively correlated with NK cells, CD8^+^ T cells, and M1 macrophages
TIME-associated biomarkers				
TILs	Clinical trial	PD-1	CRR, PFS, pCR; Paradoxical	TILs plays both positive and negative roles in executing anti-tumor immunity, depending on their types, density, proportion, and gene expression profiles
TCF-1^+^ T cells	Clinical trial; Clinical samples; Cell lines; Mouse models	PD-1; CTLA-4	Improved pCR	1) TCF-1^+^ CD8^+^ T cells will proliferate, self-renew and differentiate into TCF-1^-^ CD8^+^ T cells after treated with ICIs; 2) TCF-1 is essential for the stem-like function of CD8^+^ T cells and intratumoral immune response
TAMs	Clinical trial; Clinical samples; Cell lines; Bioinformatics analysis	PD-1	PD-L1^-^ TAMs: Improved OS; Improved PFS Reduced density of M2-TAMs: Improved MPR	1) The distance between PD-L1^-^ TAMs and tumor cells is closer; 2) TAMs secrete chemokines such as CXCL5 and CCL18 to induce immune suppression and facilitate tumor proliferation; 3) TAMs are associated with the level of TMB and PD-L1 expression
Cytokines	Clinical trial; Clinical samples; Cell lines; Bioinformatics analysis	PD-1; PD-L1	Pathologic or clinical response; Paradoxical	1) IFN-γ-related genes are associated with antigen presentation, cytotoxicity, and chemokines secretion; 2) TAMs release CXCL5 to recruit MDSCs to TME via CXCR2-CXCL5 axis and release CCL18 to promote tumor proliferation via JAK2/STAT3 pathway; 3) CCL19 activates MEK1-ERK1/2 and PI3K-AKT pathway in M1-TAMs by CCR7 to mediate their chemotaxis; 4) CCL2-CCR2 axis mediates TAMs infiltration to affect PD-1 signaling in cancer cells, leading to their immune evasion
Host-associated biomarkers				
PNI	Clinical data;	PD-1	Improved OS; Improved PFS	1) Low albumin concentration reflects malnutrition status, which is regarded as a negative prognostic factor; 2) Lymphocytes play a predominant role in immune surveillance against tumor cells
NLR	Clinical trial; Clinical data	PD-1	Unfavorable OS; Unfavorable PFS	1) Neutrophils undermine anti-tumor immunity mediated by NK cells and T cells; 2) Cytokines (e.g., IL-1, IL-6) released by neutrophils promote tumor progression
ATB use	Clinical data; Meta-analysis	PD-1; PD-L1; CTLA-4	Unfavorable OS	Both ATB and ICIs treatment abate the amount and diversity of microbiomes. Microbiomes are conducive to antigen presentation and effector T cells’ recruitment and activation
Endogenous glucocorticoid	Clinical data; Clinical samples; Cell lines; Mouse models	PD-1; PD-L1	Unfavorable OS; Unfavorable PFS; Unfavorable ORR	1) Elevated glucocorticoid is associated with lower infiltration level of CD4^+^ T cells and CD8^+^ T cells, as well as higher NLR; 2) More active glucocorticoid suppresses IFN-γ secretion from CD8^+^ T cells and curbs inflammatory state of TAMs; 3) GR downregulates MHC-1 level of cancer cells and inhibits the infiltration of cytotoxic T cells
ctDNA	Clinical trial	PD-1; LAG-3	Unfavorable OS; Unfavorable PFS; Unfavorable cCR	ctDNA clearance represents neoantigen-specific T cell responses and expansions

APCs, antigen-presenting cells; CBR, clinical benefit rate; cCR, clinical complete response; CRR, complete response rate; CTLs, cytotoxic lymphocytes; DCR, disease control rate; GR, glucocorticoid receptor; LAG-3, lymphocyte activation gene-3; MDSCs, myeloid-derived suppressor cells; MPR, major pathologic response; pCR, pathologic complete response.

*Prognostic value refers to the predictive value of biomarkers when their expression, level or density are elevated unless otherwise specified.

In addition to summarizing well-established biomarkers such as PD-L1, TMB, MSI-H, and TILs, some relatively novel markers are also discussed in this review, including DNA methylation, amplification of chromosome 11q13 and specific genes, and cytokines’ levels. We found that different studies have yielded conflicting conclusions regarding the prognostic significance of certain biomarker. Regarding PD-L1, many clinical studies failed to establish a statistically significant association between its expression and the efficacy of ICIs (**Table 1**). Similarly, it is also reported that TMB is insufficient to predict the outcomes of immunotherapy (31). These discrepancies might be attributed to the differences in detection methods, cut-off points selection, sample sizes, and treatment patterns. Recently a team has developed copy number alteration (CNA)-corrected TMB to predict the efficacy of PD-1 inhibitors plus chemotherapy (124), suggesting that biomarker standardization and adjustment warrants further exploration. The timing of detecting biomarkers’ content and the attention to their dynamic changes are also crucial. When baseline levels of a biomarker are unrelated to efficacy, positive conclusions may be drawn from its post-treatment level, or from its dynamic alteration during therapy.

We have also summarized potential biomarkers for EC immunotherapy in this section based on the quality of evidence (**Table 4**). They fit into one of the following two features: 1) Although confirmed as associated with therapeutic efficacy in EC, evidence sources encompass all treatment methods, and are not confined to immunotherapy. Additionally, these markers affect prognosis through mechanisms involving the TIME or anti-tumor immunity; 2) Markers reported to be associated with the prognosis of immunotherapy in other types of cancers, primarily lung cancer and gastrointestinal cancers, while relevant research in EC is limited. We chose gastrointestinal tumors as a reference, since the esophagus and other gastrointestinal organs share similar histological structures and embryological origins. Lung cancer was also selected, both because of the anatomical proximity of the lungs and esophagus and considering the fact that studies of lung cancer are relatively abundant and advanced. There is a current trend in oncology research of validating results derived from lung cancer studies in other types of cancers.

**Table 4:** Prognostic value of potential biomarkers with elevated levels.

Biomarker	Source of evidence	Prognostic value	Possible mechanism	Ref.
Tumor-cell-associated biomarkers				
SOCS3	Bioinformatics analysis	Unfavorable OS	1) SOCS3 promotes the infiltration of CAFs, M2-TAMs, and Tregs; 2) SOCS3 methylation is negatively related to the dysfunction of T cells	(125)
METTL3	Bioinformatics analysis	Unfavorable OS; Unfavorable PFS; Unfavorable DFS	METTL3 correlates with immune genes and the infiltration of B cells, effector memory CD8^+^ T cells, macrophages, NK cells and neutrophils	(126–128)
B7-H3	Clinical samples	Unfavorable OS	B7-H3 positively correlates with the infiltration of Foxp3^+^ Tregs and CD68^+^ macrophages and negatively correlates with the density of CD3^+^ T cells and CD8^+^ T cells in TME	(129, 130)
TIME-associated biomarkers				
LAG-3	Clinical trial; Clinical samples	Improved OS; Improved PFS; Unfavorable RFS; Paradoxical	1) LAG-3 level is positively correlated with the density of CD3^+^ TILs, CD4^+^ TILs and CD8^+^ TILs, and the ratio of CD4^+^/CD8^+^ TILs, indicating an inflammatory TIME; 2) LAG-3 is co-expressed with other immune checkpoints such as CTLA-4	(131–134)
TIM-3	Clinical samples; Bioinformatics analysis	Unfavorable OS	1) TIM-3 inhibits the function of CTLs and effector Th1 cells; 2) TIM-3 plays a role in the generation and differentiation of MDSCs; 3) TIM-3 suggests an augmented activity and apoptosis of Foxp3^+^ Tregs	(135, 136)
TIGIT	Meta-analysis; Clinical samples; Cell lines; Bioinformatics analysis	Unfavorable OS; Unfavorable PFS	1) By biding to CD155, TIGIT suppresses the secretion of IL-12 and IFN-γ and stimulates the release of IL-10 in DC cells via ERK pathway; 2) TIGIT competes with the co-stimulatory receptor CD226 for binding to CD155, thereby inducing CD8^+^ T cell exhaustion; 3) TIGIT^+^ Tregs inhibit the function of Th1 and Th17 cells by IL-10 and fgl2	(136–139)
VISTA	Clinical samples	Favorable OS	1) VISTA pathway attenuates the level of cytokines including IL-2, IL-17, IFN-γ, CCL5; 2) VISTA is co-expressed with CD4 and CD68 in TILs	(140, 141)
MDSCs	Clinical samples; Cell lines; Mouse models	Unfavorable OS	1) MDSCs impair the function and proliferation of T cells and induce their apoptosis by arginase1, iNOS, IDO, HO-1, and NOX2; 2) MDSCs stimulate the proliferation and infiltration of Tregs by secreting IL-10 and IFN-γ; 3) CD14^+^ HLA-DR^-/low^ MDSCs exhibit higher expression of PD-L1 to suppress T cell proliferation	(142, 143)
Host-associated biomarkers				
Obesity	Meta-analysis; Clinical data; Mouse models	Improved OS; Improved PFS; Paradoxical	Adipose tissue releases leptin, TNF-α, IL-6, leading to dysfunction and exhaustion of immune cells, including elevated PD-1 expression on T cells, undermined NK cells and imbalance of the ratio of M1/M2 macrophages	(144, 145)
CCS	Clinical samples; Clinical data; Cell lines; Mouse models	Unfavorable OS; Unfavorable PFS; Unfavorable DCR	CCS is induced by a wide range of cytokines secreted by tumor or immune cells, including TNF-α, IL-1, IL-6, IL-8, TGF-β, which are considered negative factors for anti-tumor immunity. They lead to exhausted T cells, impaired NK cells and DCs, accumulated MDSCs, and increased Tregs and glucocorticoid level	(146, 147)
Distress	Meta-analysis; Clinical trial; Clinical samples	Unfavorable PFS; Unfavorable ORR Unfavorable DCR	1) Distress represents an activated HPA axis, leading to an elevated level of glucocorticoid, which is an adverse prognostic biomarker; 2) Depressive patients exhibit an enhanced COX-2-PGE2 axis, inducing the infiltration of MDSCs, Tregs and M2-TAMs; 3) Depressive patients show more MDSCs recruited by neuropeptide Y, less Tregs caused by scanty IL-2, and reduced CD8^+^ T cells	(148–151)

B7-H3, B7 homologue 3; CAFs, cancer-associated fibroblasts; CCS, cancer cachexia syndrome; COX-2, cyclooxygenase-2; CTLs, cytotoxic T lymphocytes; DCR, disease control rate; DFS, disease-free survival; HPA, hypothalamus–pituitary–adrenal; MDSCs, myeloid-derived suppressor cells; METTL3, methyltransferase-like 3; PGE2, prostaglandin E2; RFS, recurrence-free survival; SOSC3, the suppressor of cytokine signaling 3; TGF-β, transforming growth factor-β; TIGIT, T cell immunoglobulin and ITIM domain; TNF-α, tumor necrosis factor-α; VISTA, V-domain Ig suppressor of T cell activation.

**Figure 2:**
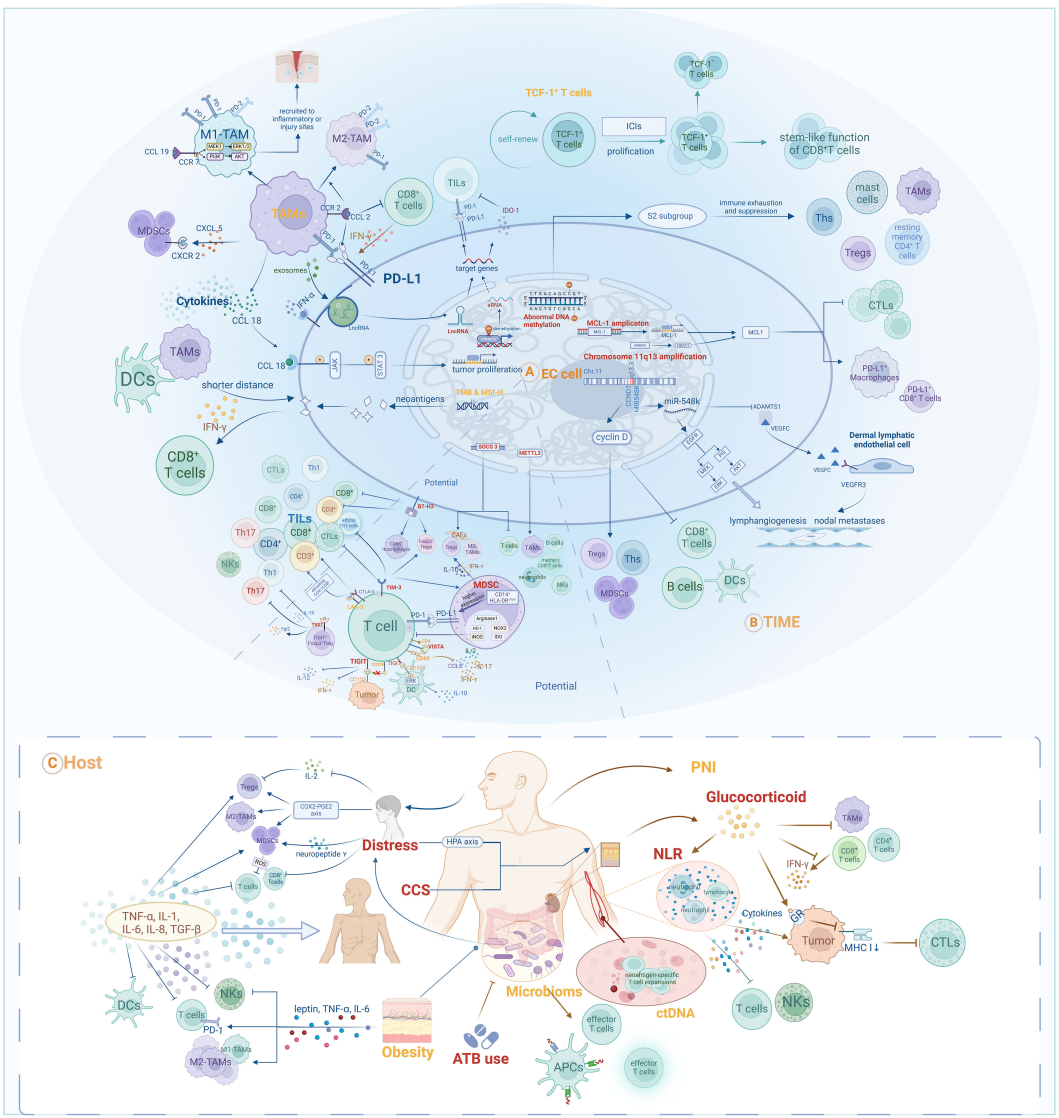
Prognostic and potential biomarkers for immunotherapy in EC and their interactive mechanisms: **(A)** Tumor-cell-associated biomarkers; **(B)** TIME-associated biomarkers; **(C)** Host-associated biomarkers.

Biomarkers summarized in the main sections of this review and potential biomarkers included in the Discussion section are illustrated in **Figure 2**. We found that various of these biomarkers can mutually interact to influence anti-tumor responses during immunotherapy. For instance, multiple biomarkers ultimately impact the responses of EC patients by mediating the quantity, activity, or phenotype of TILs. TAMs both influence the TIME by secreting chemokines to recruit MDSCs, and can also be modulated by chemokines, thereby assisting EC cells in evading anti-tumor immune responses (85, 86, 89, 91). These findings indicate that further research into the mechanisms underlying the activities of various biomarkers and to delineate their interactions will be of vital importance to provide comprehensive understanding of prognostic biomarkers in the context of EC immunotherapy.
